# PbCOP1.1 Contributes to the Negative Regulation of Anthocyanin Biosynthesis in Pear

**DOI:** 10.3390/plants8020039

**Published:** 2019-02-12

**Authors:** Meng Wu, Min Si, Xieyu Li, Linyan Song, Jianlong Liu, Rui Zhai, Liu Cong, Rongrong Yue, Chengquan Yang, Fengwang Ma, Lingfei Xu, Zhigang Wang

**Affiliations:** College of Horticulture, Northwest A&F University, Taicheng Road NO.3, Yangling 712100, China; wumeng530@nwafu.edu.cn (M.W.); siminvery6@163.com (M.S.); lixieyu@nwafu.edu.com (X.L.); linyans@yeah.net (L.S.); pearliu@nwafu.edu.cn (J.L.); right_1989313@163.com (R.Z.); imcongliu@163.com (L.C.); yuerongrong@nwafu.edu.cn (R.Y.); cqyang@nwsuaf.edu.cn (C.Y.); fwm64@nwsuaf.edu.cn (F.M.)

**Keywords:** pear, PbCOP1, anthocyanin, transient expression, color fading

## Abstract

The synthesis of anthocyanin in pear (*Pyrus bretschneideri*) fruit is regulated by light. However, little is known about the molecular mechanisms of pear fruit coloring mediated by upstream light-signaling regulators. Here, the photoresponse factors CONSTITUTIVE PHOTOMORPHOGENIC (COP) 1.1 and 1.2 were cloned from ‘Red Zaosu’ peel to study their functions in pear fruit coloring. The overexpression vectors pBI121-PbCOP1.1 and pBI121-PbCOP1.2 were constructed to analyze their effects on anthocyanin synthesis in pear fruit. A protein sequence alignment and phylogenetic tree analysis revealed that PbCOP1 proteins are highly homologous with those of other species. An analysis of tissue differential expression showed that the greatest expression levels of PbCOP1s occurred in the leaves. Their expression levels increased in the leaves during development, when the leaves changed from red to green. The overexpression of PbCOP1s in the peel resulted in reduced anthocyanin synthesis at the injection sites. A quantitative PCR analysis of the injection sites showed that PbCOP1.1 significantly inhibited the expression of the anthocyanin synthesis-related genes CHI, DFR, UFGT2, bHLH3, HY5 and GST. Based on the above results, we hypothesize that PbCOP1.1 is an anthocyanin synthetic inhibitory factor of pear coloration.

## 1. Introduction

Fruit color is an important factor that influences consumer choice. Red-skinned pear is popular among consumers because of the bright color. Globally, European pear contains the most red-skinned cultivars, and some, like Red Bartlett, undergo color fading after ripening. This phenomenon has serious effects on the economic values of these cultivars. Anthocyanins are the main pigments that determine pear fruit coloration [1]. Consequently, it is important to study the mechanisms of anthocyanin accumulation and degradation in pear.

Anthocyanin biosynthesis is regulated by structural (*PAL*, *CHS*, *CHI*, *F3H*, *DFR*, *ANS*, and *UFGT*) and regulatory genes [2,3,4,5]. The latter can act as master regulators to coordinate the expression of the former in the anthocyanin biosynthetic pathway. Regulatory genes are mainly derived from two major classes of transcription factors, the myeloblastosis (MYB) and basic helix–loop–helix (bHLH) families, which, along with WD-repeat protein, form a MYB–bHLH–WDR transcription complex to regulate anthocyanin synthesis [6,7]. In addition, anthocyanin synthesis is controlled by external environmental factors, such as nutrient depletion, drought, pathogen infection, temperature, and light [8]. Light is a decisive environmental factor that affects the accumulation of anthocyanins in fruit. Light can cause an increase in anthocyanin abundance, indicating that there is a correlation between anthocyanin synthesis and the status of the light-signaling pathway [9,10,11,12].

Higher plants have sophisticated light-receiving and signal transduction systems. The E3 ubiquitin ligase COP1 is a photomorphogenic inhibitor that plays a decisive role in phototransduction and acts as a molecular switch during light-induced plant growth and development. It affects flowering time, photoperiodic growth and stomatal development [13,14,15]. CONSTITUTIVELY PHOTOMORPHOGENIC1/SUPPRESSOR OF PHYA-105 (COP1/SPA) ubiquitin ligase degrades the photomorphogenesis-promoting transcription factors ELONGATED HYPOCOTYL5 (HY5), LONG AFTER FAR-RED1 and LONG HYPOCOTYL IN FAR-RED1 through the 26S proteasome pathway [16,17,18]. In addition to these transcription factors, the photoreceptors phytochrome (PHY) A and B and cryptochrome (CRY) 2 are subjected to COP1-mediated degradation [19,20,21,22]. Among these transcription factors, HY5 positively regulates anthocyanin synthesis by binding to the promoters of anthocyanin biosynthetic genes. COP1 degrades HY5 by ubiquitination to reduce anthocyanin accumulation [23,24]. In pear, the blue-light signal transduction module CRY–COP1–HY5 is involved in the regulation of anthocyanin synthesis, but this does not directly verify the function of COP1 [25]. In apples and Arabidopsis, the COP1/SPA complex regulates anthocyanin synthesis by regulating the transcriptional and translational levels of MYB transcription factors [26,27].

COP1 plays an important role in the regulatory pathways of anthocyanin synthesis. At present, no functional verification study has proven that *PbCOP1* effects the coloration of pear fruit. In this study, two *COP1* genes, *PbCOP1.1* and *PbCOP1.2*, were isolated from the fruit of ‘Red Zaosu’. The tissue-specific expression, and expression at different leaf developmental stages for *PbCOP1s* was investigated. It indicates that *PbCOP1s* may also inhibit anthocyanin synthesis in pears. We overexpressed *PbCOP1s* to identify its function in pear fruits, and then the structural and regulatory genes of anthocyanin synthesis pathway regulated by COP1 was discussed. 

## 2. Results

### 2.1. PbCOP1 Cloning and Homology Analysis

**Figure 1:**
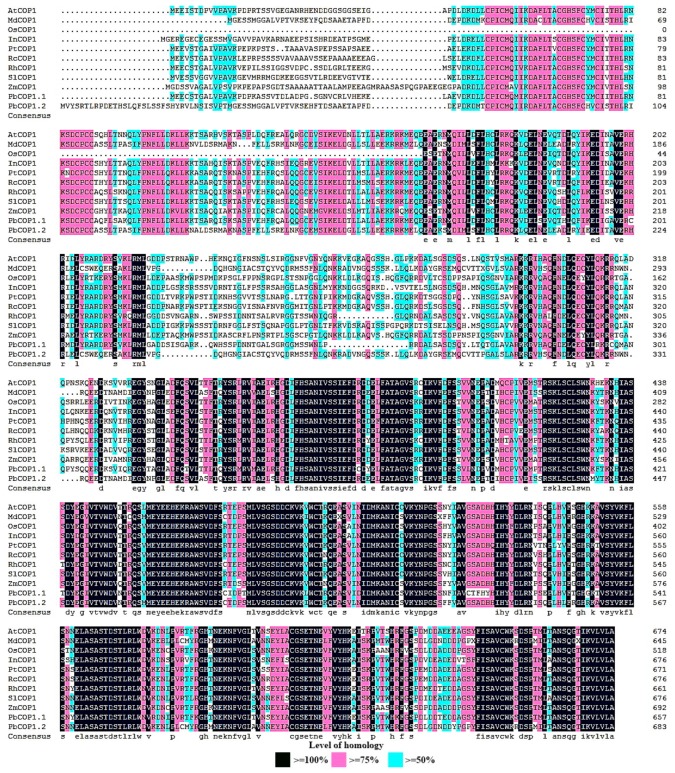
Amino acid sequence alignment analysis of COP1 in pear and other related plants. At, *Arabidopsis thaliana*; Md, *Malus × domestica*; Os, *Oryza sativa* subsp. *indica*; In, *Ipomoea tricolor*; Pt, *Populustrichocarpa*; Rc, *Ricinus communis*; Rh, *Rosa* spp. hybrid cultivar; Sl, *Solanum lycopersicum*; Zm, *Zea mays*; Pb, *Pyrus bretschneideri.*

PbCOP1 genes were isolated from pear. A BLAST-algorithm based search of the pear genomic database found two COP1-like genes, named PbCOP1.1 and PbCOP1.2, respectively. We cloned the full-length cDNA sequences of PbCOP1.1 and PbCOP1.2 (Supplemental Figure S1). The open reading frame of PbCOP1.1 is 2055 bp and encodes a protein containing 684 amino acid residues. The open reading frame of PbCOP1.2 is 1977 bp and encodes a protein containing 658 amino acid residues. The amino acid sequences of the predicted PbCOP1.1 and PbCOP1.2 proteins were highly similar (60.23%). A multiple COP1 protein sequence alignment indicated that PbCOP1s were highly homologous with those of other species, including Arabidopsis thaliana AtCOP1 (AEC08766.1), Malus × domestica MdCOP1 (AB668570.1), Oryza sativa subsp. indica OsCOP1 (BAA94422.1), Ipomoea tricolor InCOP1 (AAG31173.1), Populustrichocarpa PtCOP1 (XP_002321154.1), Solanum lycopersicum SlCOP1 (AAC98912.1), Ricinus communis RcCOP1 (XP_002534127.1), Rosa spp. hybrid cultivar RhCOP1 (AAK81856.1), and Zea mays ZmCOP1 (ACG47820.1) (Figure 1).

**Figure 2:**
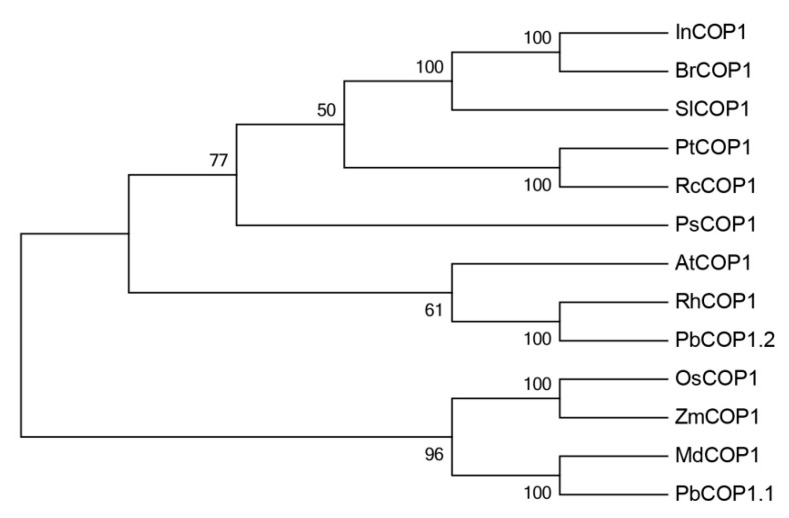
Phylogenetic tree of COP1 from different species. In, *Ipomoea tricolor* (AAG31173.1); Br, *Brassica capitata* (AAN86553.1); Sl, *Solanum lycopersicum*(AAC98912.1); Pt, *Populustrichocarpa* (XP_002321154.1); Rc, *Ricinus communis* (XP_002534127.1); Ps, *Pisum sativum* (CAB94800.1); At, *Arabidopsis thaliana* (AEC08766.1); Rh, *Rosa* spp. hybrid cultivar (AAK81856.1); Pb, *Pyrus bretschneideri* (XP_009357519.2, XP_009340196.1); Os, *Oryza sativa* subsp. *indica* (BAA94422.1); Zm, *Zea mays* (ACG47820.1); Md, *Malus × domestica* (AB668570.1).

Furthermore, a phylogenetic analysis based on amino acid sequences was used to analyze the phylogenetic relationship between each of the PbCOP1s and the COP1s from other plant species. PbCOP1.1 was most closely related with MdCOP1, and they form one clade. Interestingly, PbCOP1.2 was most similar to RhCOP1 and AtCOP1, and they clustered together in the other clade (Figure 2).

### 2.2. Expression Level Analysis of PbCOP1s in Pear

**Figure 3:**
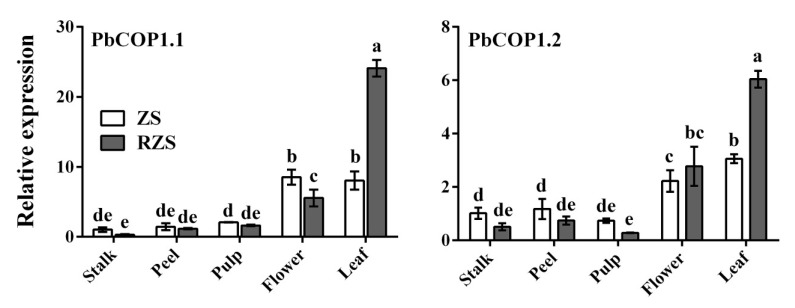
Expression patterns of *PbCOP1.1* and *PbCOP1.2* in different pear tissues. ZS, ‘Zaosu’, RZS, ‘Red Zaosu’. All data are presented as the means ± standard errors (SE) of three biological replicates, and different letters above the columns indicate significant differences at P < 0.05 (Duncan’s range test).

*PbCOP1.1* and *PbCOP1.2* were expressed in stalks, peel, flesh, flowers and mature leaves, with the greatest expression level occurring in mature leaves, followed by flowers. The *PbCOP1.1* expression level was lowest in the stalk, and the *PbCOP1.2* expression level was lowest in the pulp. In mature leaves, the *PbCOP1.1* and *PbCOP1.2* expression levels in ‘Red Zaosu’ were more than twice as high as in ‘Zaosu’ (Figure 3). 

**Figure 4:**
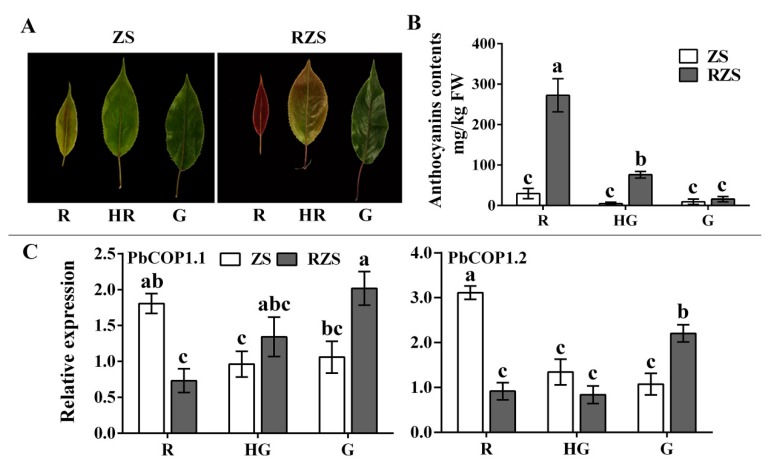
Phenotypes, anthocyanin contents and expression levels of *PbCOP1* genes at different leaf developmental stages. (**A**) Leaf phenotypes. (**B**) Anthocyanin contents in leaves. (**C**) *PbCOP1* expression levels in leaves. R, red; HR, half red; G, green. ZS, ‘Zaosu’; RZS, ‘Red Zaosu’. All data are presented as the means ± standard errors of three biological replicates, and different letters above the columns indicate significant differences at P < 0.05 (Duncan’s range test).

Subsequently, we analyzed the *PbCOP1*s’ expression levels in the leaves of ‘Red Zaosu’ and ‘Zaosu’ at different developmental stages. ‘Red Zaosu’ is a red sport of ‘Zaosu’. The anthocyanin contents of ‘Red Zaosu’ decreased sharply as leaves changed color from fully red to fully green. The anthocyanin contents of ‘Zaosu’ were slightly reduced, and there were no significant differences among the developmental stages (Figure 4A,B). The *PbCOP1.1* expression level in fully green leaves was approximately three times of that in fully red leaves. The *PbCOP1.2* expression level in fully green leaves was approximately two times that in fully red leaves. As the leaves of ‘Red Zaosu’ changed from fully red to fully green, the expression levels of *PbCOP1.1* and *PbCOP1.2* gradually trended upward, which was the opposite of the anthocyanin accumulation pattern. However, *PbCOP1.1* and *PbCOP1.2* showed downward expression trends in ‘Zaosu’ during the same developmental period (Figure 4C). This indicated that *PbCOP1.1* and *PbCOP1.2* may be negatively regulate anthocyanin synthesis. In addition, we also examined the anthocyanin contents and the expression levels of *PbCOP1s* of ‘Red Bartlett’ at different stages of fruit development. The anthocyanin contents showed a rise–drop tendency. *PbCOP1.1*’s expression level showed a drop–rise tendency, which was the opposite of the anthocyanin. However, the *PbCOP1.2*’s expression level was on the rise (Supplementary Material Figure S2). This suggests that *PbCOP1.1* and *PbCOP1.2* may have different functions in the peel.

### 2.3. PbCOP1.1 Negatively Regulates Fruit Coloration in Pear

**Figure 5:**
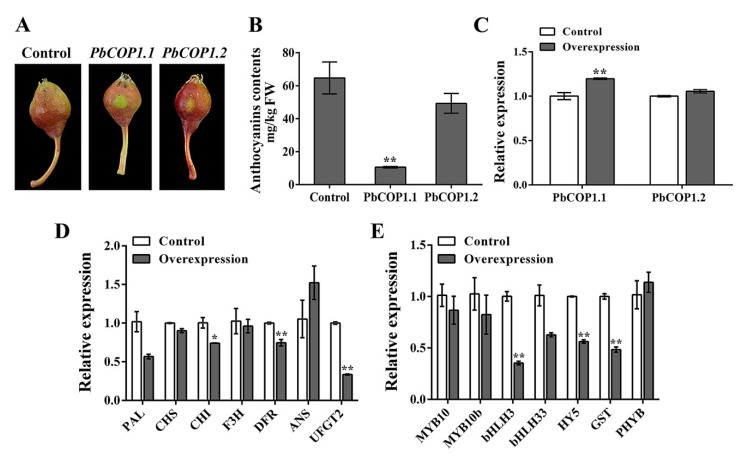
The overexpression of *PbCOP1.1* reduced fruit coloring in the ‘Red Bartlett’ peel. (**A**) The ‘Red Bartlett’ peel coloration around the injection sites after overexpressing *PbCOP1s.* Seven days after injection, the peels around the injection sites were used for qRT-PCR and anthocyanin contents analysis. (**B**) The anthocyanin contents in ‘Red Bartlett’ peels around the injection sites. (**C**) Expression levels of *PbCOP1*s in ‘Red Bartlett’ peels around the injection sites. (**D**) Expression levels of structural genes in *PbCOP1.1* overexpressed fruit. (**E**) Expression levels of regulatory and anthocyanin-related synthetic genes in *PbCOP1.1* overexpressed fruit. Error bars indicate the standard errors of the means. * indicates differences that are statistically significant at the P < 0.05 level; ** indicates differences that are statistically significant at the P < 0.01 level.

To verify *PbCOP1* functions in pear fruit coloration, their expression levels were enhanced in transient overexpression assays. The overexpression of both *PbCOP1.1* and *PbCOP1.2* inhibited fruit coloration around the injection sites in ‘Red Bartlett”. The expression level of *PbCOP1.1* was significantly enhanced in its overexpressing fruit compared with in the empty vector, while that of *PbCOP1.2* was also enhanced in its overexpressing fruit, but not significantly. Compared with the control, the anthocyanin contents were reduced significantly in the peel of fruit overexpressing *PbCOP1.1*, while no significant difference was observed in the anthocyanin contents of fruit overexpressing *PbCOP1.2* (Figure 5A–C). Therefore, the expression levels of anthocyanin-related synthetic genes were assessed by real-time PCR using *PbCOP1.1* overexpressing peel.

The expression levels of structural genes were down-regulated, and among them *CHI*, *DFR* and *UFGT2* were significantly down-regulated. Although *ANS* was up-regulated, no significant changes were detected in its expression levels (Figure 5D). Moreover, the expression levels of the regulatory genes *(bHLH3, HY5 and GST)* in anthocyanin synthesis pathway were significantly down-regulated. The expression level of the light-responsive gene *PHYB* did not show any significant differences (Figure 5E). Thus, the overexpression of *PbCOP1.1* decreased the expression levels of anthocyanin-related synthetic genes and negatively regulated fruit coloration in pear.

## 3. Discussion

In plants, *COP1* is an inhibitor of photomorphogenesis, interacting with upstream photoreceptors and downstream target proteins to regulate light-induced plant growth and development [28]. When the cells are located in the dark, *COP1* is localized to the nucleus and the photomorphogenesis is inhibited. But when the cells are exposed to light, *COP1* is transferred to the cytoplasm and photomorphogenesis is restored [29]. Light and photoreceptor proteins regulate the nuclear localization of *COP1* proteins. These photoreceptors mainly include PHYs, CRYs and UV-B receptors [30,31,32]. Photoreceptors regulate the *COP1* protein content in the nucleus in different ways. *PHYA* may inhibit the transport of the *COP1* protein from the nucleus into the cytoplasm. *PHYB* mainly regulates the transport or degradation of *COP1* proteins in the nucleus [33]. *COP1* regulates anthocyanin synthesis by altering its sub-cellular localization. First, HY5 protein was degraded through *COP1/SPA* E3 ubiquitin ligase in dark-growing seedlings. HY5 promotes the biosynthesis of anthocyanins by inducing the expression of anthocyanin biosynthetic genes such as *CHS*, *DFR*, *ANS* and *PAP1* [16,23,34]. Second, the *COP1* protein promotes MYB protein degradation through ubiquitination, resulting in the down-regulation of the main structural genes and a decrease in the anthocyanin contents [26,27,35]. Other studies have shown that, *COP1* does not inhibit anthocyanin accumulation in the nucleus. Under dark conditions, the *COP1* content of ‘Jingxiu’ grapefruit increases compared with under light conditions, and the anthocyanin contents decreases. However, in another study, no differences were found in the *COP1* contents and anthocyanin abundances between ‘Jingyan’ grapes maintained under light and dark conditions [36]. In this study, the analysis of the phylogenetic tree’s cluster showed that *PbCOP1.1* and *MdCOP1* formed one clade, while *PbCOP1.2, AtCOP1* and *RhCOP1* were assigned to another one (Figure 2). *COP1* negatively regulates the accumulation of anthocyanin in Arabidopsis and apple in the darkness; therefore, *PbCOP1.1* and *PbCOP1.2* may be involved in the regulation of anthocyanin synthesis. In ‘Red Zaosu’, the transcriptional levels of the *PbCOP1*s were much greater in fully red leaves compared with fully green leaves, which was contrary to the anthocyanin accumulation level (Figure 4). This may indicate that *PbCOP1*s negatively regulate anthocyanin synthesis. In addition, *COP1* was considered a candidate gene in a transcriptome study in which genes related to color fading in ‘Red Bartlett’ were screened [37]. Therefore, we selected ‘Red Bartlett’ as the material used in the functional verification of *PbCOP1*s. 

Here, the expression levels of *PbCOP1.1* in the peel around injection areas increased and the anthocyanin levels decreased significantly, when compared with the empty vector. However, the *PbCOP1.2* expression levels were only slightly elevated, and the anthocyanin levels decreased slightly. This may result from the presence of other mechanisms that inhibit the overexpression of *PbCOP1.2*, or due to *PbCOP1.2* not acting as an efficient gene in the anthocyanin synthetic pathway in pear fruit. After overexpressing *PbCOP1.1*, the expression levels of anthocyanin synthesis-related genes, except for *ANS*, decreased to different degrees, and *CHI*, *DFR*, *UFGT2*, *bHLH3*, *HY5* and *GST* were significantly down-regulated (Figure 5). This further demonstrates that *PbCOP1.1* negatively regulates the synthesis of anthocyanin involved in the coloration of pear fruit, as reported previously [26,27]. Indeed, *MYB10* and *MYB10b* act as positive regulators to induce the expression levels of structural genes in the anthocyanin biosynthetic pathway in pear [38,39,40]. *PbCOP1* and *PbMYB10* interact physically with each other [25]. Moreover, the *MdCOP1* protein directly interacts with the *MdMYB1* protein, resulting in the degradation of *MdMYB1*. However, the overexpression of *MdCOP1* in apple fruit revealed that *MdMYB1* was significantly different only at the protein, and not at the transcriptional level [26]. This may be why *MYB10* is not significantly down-regulated at the transcriptional level after the overexpression of *PbCOP1.1*. In pear, the overexpression of *MYB10* causes the up-regulation of the structural gene *UFGT*, and the overexpression of *MYB10b* promotes the up-regulation of *DFR* [39,40]. The overexpression of *MYB10/bHLH3* in peach induced the expression levels of structural genes *CHS*, *DFR* and *UFGT* [41]. GST proteins are involved in the transport of anthocyanins and positively regulate the accumulation of anthocyanins [42,43,44]. In this study, the overexpression of *PbCOP1.1* caused an enhanced degradation of the anthocyanin contents, possibly because *PbCOP1.1* degraded *HY5* and *MYB10* proteins through ubiquitination, resulting in the down-regulated expression levels of *CHI*, *DFR*, *UFGT2*, *bHLH3* and *GST*. 

*COP1* can degrade *MYB* and *HY5* proteins by ubiquitination, thus directly or indirectly affecting the expression levels of anthocyanin-related synthetic genes that regulate anthocyanin accumulation in plants. However, no studies have determined whether *COP1* can directly act on the promoters of the structural genes involved in anthocyanin synthesis. Studies on the regulatory mechanisms of *COP1* in anthocyanin synthesis have focused on Arabidopsis and apple, but the specific regulatory mechanisms remain unclear. Among consumers, fruit color is treated as an indicator of fruit quality, but there are few studies on the roles of *COP1*s in fruit coloring. The results of this study indicated that the overexpression of *PbCOP1.1* in ‘Red Bartlett’ peel negatively regulated the anthocyanin synthesis, resulting in the down-regulated expression of the anthocyanin-related genes *CHI*, *DFR*, *UFGT2*, *bHLH3*, *HY5* and *GST*. This provides a theoretical reference for understanding the molecular mechanisms of the light-signaling molecule *PbCOP1* that are involved in regulating the anthocyanin-related synthetic genes to affect the coloration of pear fruit. 

## 4. Materials and Methods

### 4.1. Plant Materials, Treatments and Growth Conditions

The leaves and fruit of ‘Red Zaosu’ and ‘Zaosu’ were collected from an orchard in Meixian, Shaanxi Province, China, in May 2017. The young leaves of ‘Red Zaosu’ are fully red, but the color gradually fades with leaf age, becoming fully green. Leaves were picked during three growth periods represented by young (fully red), tender (half red), and mature (fully green) leaves. twenty leaves acted as biological repeats, with three biological replicates per period. The different tissues (stalks, peels, pulp, leaves and flowers) of the two cultivars were collected at 45 d after flowering and immediately frozen in liquid nitrogen, and then stored at −80 °C for further use.

The fruit of ‘Red Bartlett’ were collected from the same orchard. In April 2018, trees with strong growth were selected and their fruit were bagged in double-layered paper bags at 5 d after flowering. The bags were unpacked before being injected with *COP1*-containing constructs. The fruit were harvested 7 d after injection, and the peels near the injection areas were isolated. The peels of 10 fruits were treated as a biological repeat. The peels of the fruit were removed at about 1 mm thickness. All tissues were analyzed using three biological replicates. The fruits of ‘Red Bartlett’ were collected at 20, 50 and 80 d after flowering. 15 fruits were treated as a biological repeat. There are three biological replicates (Supplementary Material Figure S2). The fruits were immediately frozen in liquid nitrogen, and then stored at −80 °C for further use.

### 4.2. Anthocyanin Analysis

The total anthocyanin extraction was carried out as described by Giusti and Wrolstad [45], with slight modifications. Samples (0.2 g) rapidly ground into a powder in liquid nitrogen, and then 1% HCL-methanol solution (5 mL) was added. Specific extraction steps and calculation method of anthocyanin content are in accordance with the method of Wang et al. [37]. The anthocyanin content was determined spectrophotometrically. The absorbance of each extract was measured at 520 nm and 700 nm with a UV-Visible spectrophotometer (UV-1700, Kyoto, Japan). The total anthocyanin content was expressed as mg/kg FW. The value used for each sample was the mean of three independent biological replicates. 

### 4.3. PbCOP1.1 and PbCOP1.2 Cloning 

Total RNA was isolated from pear cultivar ‘Red Zaosu’ fruit. RNA was extracted using the RNAprep Pure Plant Kit (Tiangen, Beijing, China) according to the manufacturer’s instructions. The RNA concentration and quality were detected by UV-Visible spectrophotometer (UV-1700, Kyoto, Japan). the First-strand cDNA was synthesized using the PrimeScript RT reagent kit with gDNA Eraser (TaKaRa, Dalian, China). The complete coding DNA sequences (CDS) of *PbCOP1* genes were isolated from the database of the pear genome (http://peargenome.njau.edu.cn/) [46]. The complete CDS were used as query to search for homologous sequences using National Center for Biotechnology Information, the GenBank accessions of *COP1* genes are *PbCOP1.1* (XP_009357519.2) and *PbCOP1.2* (XP_009340196.1). We used Primer 5.0 software to design specific primer pairs (Supplemental Table S1) for cloning. 

### 4.4. Construction of the Phylogenetic Tree

Multiple alignments of amino acid sequences were performed between pear and other plants using ClustalW in DNAMAN (LynnonBiosoft, CA, USA). The phylogenetic analysis was carried out by the Neighbor–Joining method with the JTT+G model using the MEGA 5.0 program. Branches corresponding to partitions were reproduced from 1,000 bootstrap replicates. The evolutionary distances were computed using the p-distance method. 

### 4.5. Vector Construction and Genetic Transformation

The complete cDNAs of *PbCOP1.1* and *PbCOP1.2* were cloned into the expression vector pBI121 under the control of the 35S promoter. The *pBI121-PbCOP1.1*, *pBI121-PbCOP1.2* and *pBI121* vectors were independently transformed into *Agrobacterium tumefaciens EHA105* strains that incubated in LB medium and resuspended in the infiltration buffer (10 mM MgCl_2_, 10 mM MES at pH 5.6 and 150 mM acetosyringone). Final OD_600_ was adjusted to ~0.8 before infiltration. Infiltration was conducted as described by Zhai et al. [40]. The *pBI121-PbCOP1.1-*, *pBI121-PbCOP1.2-* and negative control *pBI121*-containing bacterial solutions were independently injected 1-mm below the peel at sunset after the bags were removed of pear fruit. The fruit were harvested 7 d after injection, and the peels near the injection areas were isolated. The pear fruits infiltrated with *Agrobacterium* containing the empty *pBI121* were used as negative controls. 

### 4.6. Expression Analysis Using Quantitative Real-Time PCR (qRT-PCR)

The qRT-PCR reaction used the SYBR Premix Ex Taq II (TaKaRa), following the manufacturer’s instructions. It was performed using an Icycler iQ5 (Bio-Rad, Berkeley, CA, USA). Three biological replicates were analyzed for each sample. Each of biological samples were assayed in technical triplicates. Relative expression levels were calculated with the 2^−ΔΔCT^ algorithm method. The primers of all genes used here are provided in Supplemental Table S1**.**

### 4.7. Statistical Analysis

Data were analyzed with multivariate analysis methods using SPSS 23.0 software (SPSS, Chicago, IL, USA). An analysis of variance and significant difference tests were conducted to identify differences among means using a one-way ANOVA with Tukey’s honestly significant difference test. Drawings were constructed using GraphPad Prism 6.01 (GraphPad Prism, San Diego, CA, USA) software. 

## Supplementary Materials

The spplementary materials are available online at http://www.mdpi.com/2223-7747/8/2/39/s1.
