# Supplementary material for: PbCOP1.1 Contributes to the Negative Regulation of Anthocyanin Biosynthesis in Pear
Source: Plants (Basel). 2019 Feb 12;8(2):39. doi: 10.3390/plants8020039 (PMC6409758; doi:10.3390/plants8020039)
Supplement: Supplementary file 1 [file plants-08-00039-s001.pdf]

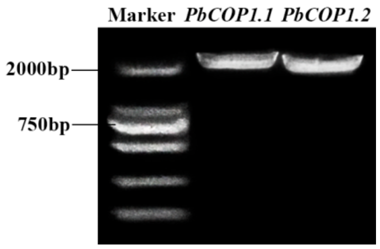

Fig. S1 The gel electrophoresis detection of *PbCOP1s* overexpression vector

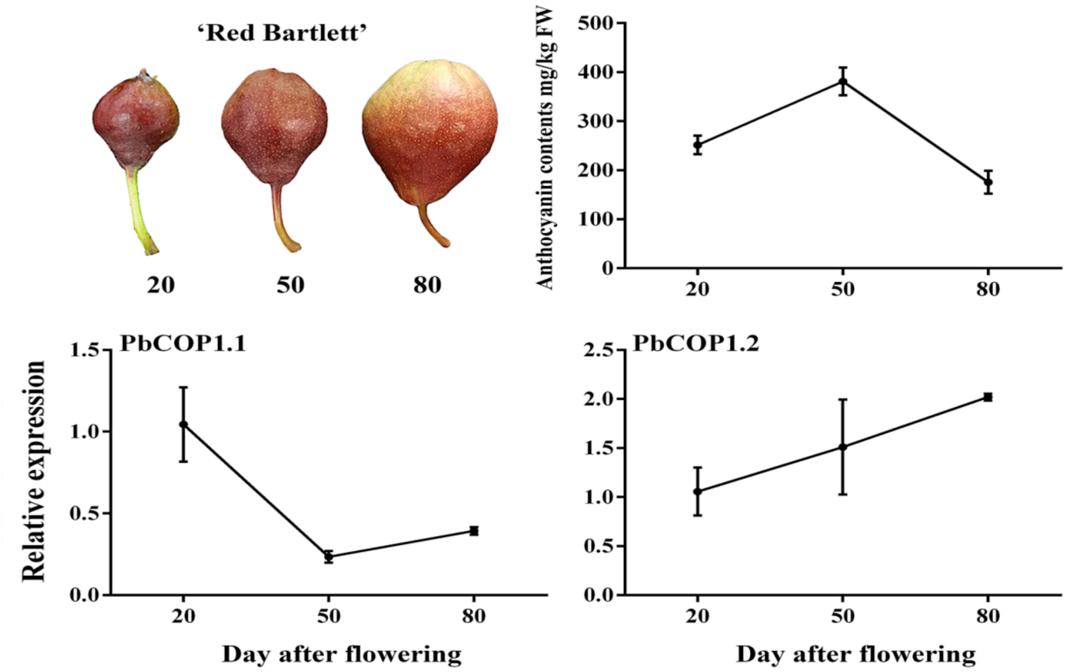

Figure S2. Anthocyanin content and *PbCOP1s* expression levels of 'Red Bartlett' at different stages of fruit development. Data are mean ± SE of three biological replicates.

Table S1 The primer sequences used for overexpression-vector construction and real-time PCR

Gene name	Forward primer sequence	Reverse primer sequence
PbCOP1.1	5'-CAATGGGTGGTGCGCTGGTC-3'	5'-GAGGAAGGCGTCTCTGATGAT-3'
PbCOP1.2	5'-CGGTTATGGAATGTGAGGGATAA-3'	5'-TTTATGCCAAGTCACAGGTTTAGAG-3'
PAL	5'-TCCATGGACAACACTCGCTT-3'	5'- AGAAACTGCAGCTCGGAACA -3'
CHS	5'-ACTGTCGAGGAAGTTCGCAA-3'	5'-CGGGATATGTGGCTTGATCC-3'
CHI	5'-ATCGAGGGCAAGTTGGTGAA-3'	5'-CCTGTGACGACGTCCTTGAT-3'
F3H	5'-TGGGTTCTGGAATGTGGCTATGGA-3'	5'-TGGGAAGACATGGATCACCGTTCA-3'
DFR	5'-GATTTCATCACCATTATCCCAACTC-3'	5'-TGCCAACTTTGACATCAACGAG-3'
ANS	5'-AGCTTCAACACCAAGTGCAAGCTC-3'	5'-TTGGATGAAGGGAGGCTGGAGAAA-3'
UFGT2	5'-GGACACTATCGGAACTCAAGG-3'	5'-AGGTCGAGTTCTTCGAAACAG-3
MYB10	5'-TCATCGACAAAGAATGGAA-3'	5'-CTAACTGAAGGCTGGGACA-3'
MYB10b	5'-AGATTTCACAGAGGACGAA-3'	5'-CCCCTAACCCTAAACTAGA-3'
bHLH3	5'-ACTTCGGCAGTGGCGTTC-3'	5'-TCACATTCTCGCGTTCAGCAA-3'
bHLH33	5'-GTGATGCAAACACTTAGGGAGC-3'	5'-TTGCCACTCACGTTATCCTTCA-3'
HY5	5'-GCGGCTGAAGAGGTTGTTGAG-3'	5'-AGCATCTGATTCTCATTCTGCAAAG
GST	5'-AAGTATGCAGGCCGTGGTC-3'	5'-CAGGTCGTTGAAGTTGTGGC-3'
PHYB	5'-ACCACAACACGGAGTCTATGAGC-3'	5'-GAATCTTTGGTGGTTTTCATGCT-3'
ACTIN	5'-CCATCCAGGCTGTTCTCTC-3'	5'-GCAAGGTCCAGACGAAGG-3'
pBI121-PbCOP1.1	5'-GGACTCTAGAGGATCCATGGTCTACTCAC GCACGC-3'	5'-GATCGGGGAAATTCGAGCTCTCAGGC TGCAAGTACGAGCAC-3'
pBI121-PbCOP1.2	5'-GGACTCTAGAGGATCCATGGAGGAGTGCT CGACCG-3'	5'-GATCGGGGAAATTCGAGCTCCTATGC CGCAAGAACCAACACT-3'
